# Cyclin D1/CDK coordination with the cellular prion protein upregulated cell proliferation signaling and preserved neurological function in acute IS rats

**DOI:** 10.7150/ijbs.98013

**Published:** 2025-09-21

**Authors:** Kun-Chen Lin, Kuan-Hung Chen, John Y. Chiang, Han-Tan Chai, Chi-Ruei Huang, Yi-Ling Chen, Yi-Ting Wang, Jun Guo, Hon-Kan Yip

**Affiliations:** 1 Department of Anesthesiology, Kaohsiung Chang Gung Memorial Hospital and Chang Gung University College of Medicine, Kaohsiung, 83301, Taiwan, R.O.C.; 2 Department of Computer Science and Engineering, National Sun Yat-Sen University, Kaohsiung 804201, Taiwan, R. O. C.; 3 Department of Healthcare Administration and Medical Informatics, Kaohsiung Medical University, Kaohsiung 807378, Taiwan, R. O. C.; 4 Division of Cardiology, Department of Internal Medicine, Kaohsiung Chang Gung Memorial Hospital and Chang Gung University College of Medicine, Kaohsiung 833401, Taiwan, R. O. C.; 5 Center for Shockwave Medicine and Tissue Engineering, Kaohsiung Chang Gung Memorial Hospital, Kaohsiung 833401, Taiwan, R. O. C.; 6 Institute for Translational Research in Biomedicine, Kaohsiung Chang Gung Memorial Hospital, Kaohsiung 833401, Taiwan, R. O. C.; 7 Department of Cardiology, The First Affiliated Hospital, Jinan University, Guangzhou 510630, China; 8 School of Medicine, College of Medicine, Chang Gung University, Taoyuan 333323, Taiwan, R. O. C.; 9 Department of Medical Research, China Medical University Hospital, China Medical University, Taichung 404333, Taiwan, R. O. C.

**Keywords:** acute ischemic stroke, cellular prion protein, cyclin D, cyclin-dependent kinase, cell proliferation signaling, cell cycle

## Abstract

We tested how the coordination between cyclin D1/cyclin-dependent kinase (CDK) and the cellular prion protein (PrP^C^) activates mitogenic/cell proliferation signaling to improve neurological outcomes in acute ischemic stroke (AIS) rats. Compared with those in adipose-derived mesenchymal stem cells (ADMSCs) and the N2a cell line, the cell viability, cell proliferation, cell-stress signaling, and wound healing rates were significantly increased upon overexpression of PrP^C^ (PrP^C-OE^) in ADMSCs (all *P<*0.001). The cell viability, proliferation. mitochondrial mass, and protein expression of mitogenic signaling markers (cyclin D1, cyclin E1, CDK2, and CDK4) were significantly increased upon PrP^C-OE^ in ADMSCs compared to ADMSCs that were subjected to a significant reversal of PrP^C-OE^ by treatment with promazine (a PrP^C^ formation inhibitor) (all *P<*0.001). After 3 h of serum-free/hypoxic conditions, the protein expression levels of cyclin D1/CDK, p-Akt and mitogenic signaling markers were significantly increased upon PrP^C-OE^ in ADMSCs compared with ADMSCs that were treated with palbociclib (a cyclin D1/CDK inhibitor). Adult male Sprague-Dawley rats (n=40) were grouped into Groups 1 (AC), 2 (AIS), 3 (AIS + ADMSCs), and 4 (AIS + ADMSCs with PrP^C-OE^). By Day 28 after AIS induction, the neurological function and numbers of NeuN+ cells and myelin basic protein (*MBP*)+ cells were lowest in Group 2, highest in Group 1 and significantly increased in Group 4 compared with Group 3, whereas the cellular levels of fibrosis and inflammation markers and protein levels of markers of apoptosis, mitochondrial and DNA damage and autophagy exhibited the opposite pattern to neurological function, and protein expression levels of cell-stress signaling proteins (PI3K, Akt, and m-TOR) and PrP^C^ progressively increased from Groups 1 to 4 (all *P<*0.0001). In conclusion, activated cyclin D1/CDK coordinated with PrP^C^ to improve neurological function in the AIS setting.

## Introduction

Ischemic stroke (IS) remains the 3^rd^ leading cause of mortality globally 1-3. Although new strategies, including the use of antiplatelet agents 4-6, the emergent use of reperfusion therapies 7, such as tissue plasminogen activator (tPA) therapy 8-10, the catheter-based endovascular removal of the thrombosis 11-14 in the larger vessels, and renewed guidelines for poststroke rehabilitation 15, 16, have reduced mortality within the last decade in patients after acute IS, the long-term comorbidity and IS-related disability and sequalae have regrettably still not improved substantially within the last 10 years 17-22. These unfavorable outcomes not only cause serious suffering for IS patients but also constitute an enormous economic burden on society. In summary, the current treatment of IS patients leaves much to be desired. This motivates the need to find a new treatment method with improved safety and efficacy for patients after IS, especially for those who are unsuitable candidates for tPA therapy or endovascular thrombectomy.

Many studies have shown that cell-based therapy has emerged as an effective therapeutic option for treating ischemia-related organ dysfunction, especially for patients who are refractory to conventional therapy 23-26. Preclinical and clinical studies have demonstrated that endothelial progenitor cells (EPCs) 25, 27 and mesenchymal stem cells (MSCs) 28-32 have therapeutic potential for treating ischemic heart disease and IS. Additionally, our animal model studies revealed that adipose-derived mesenchymal stem cells (ADMSCs) therapy or even human umbilical-derived mesenchymal stem cell therapy 28-31 markedly protected the neurological function and integrity of the brain architecture in rodents after acute IS or acute intracranial hemorrhage. The underlying mechanism 28-32 for improving the outcomes of these cell-based therapies is mainly through anti-inflammatory effects, suppression of oxidative stress and upregulation of tissue and neuronal regeneration. However, one problem is that the survival and proliferation rates of ADMSCs have been shown to be substantially diminished during ischemia, resulting in a decrease in their therapeutic effect.

Originally, investigators discovered that the cellular prion protein (PrP^C^) is a glycosylphosphatidylinositol-anchored glycoprotein that is predominantly localized in the brain and nerve cells 33, 34. Since then, PrP^C^ has been shown to act as a neuroprotective or survival protein to guard against Bcl-2-associated protein X (i.e., Bax)-mediated cell death 35. Intriguingly, our recent study 36 revealed that the overexpression of PrP^C^ (PrP^C-OE^) in ADMSCs enhanced mitochondrial biogenesis and cell proliferation and suppressed inflammatory reactions in the context of acute kidney ischemia‒reperfusion injury. However, the precise mechanism underlying the ability of PrP^C^/PrP^C-OE^ in ADMSCs therapy to increase cell proliferation and survival and reverse ischemia-related organ dysfunction is currently unclear. Interestingly, a previous study 37 revealed that cyclin D1/cyclin-dependent kinase (CDK) plays crucial roles in promoting the rate of onset and magnitude of mitogenic signaling by activating Akt. The findings from the previous study 37 were comparable with the findings of our recent study 36. However, the coordination between cyclin D1/CDK and PrP^C^ for upregulating the cell proliferation signaling and the cell cycle remains unclear. Thus, this study attempted to clarify how the coordination between cyclin D1/CDK and PrP^C^ activates cell stress and cell proliferation signaling to improve neurological outcomes in acute IS rats.

## Materials and Methods

### Ethical issues

All animal procedures were approved by the Institute of Animal Care and Use Committee at Kaohsiung Chang Gung Memorial Hospital (Affidavit of Approval of Animal Use Protocol No. 2021081802) and performed in accordance with the Guide for the Care and Use of Laboratory Animals.

The animals were housed in an Association for Assessment and Accreditation of Laboratory Animal Care International (AAALAC; Frederick, MD, USA)-approved animal facility in our hospital with a controlled temperature and light cycles (24 °C and a 12 hour light/12 dark cycle).

### Transfection of ADMSCs with plasmids for PrP^C^ expression

The methods used were described in our previous report 36. Specifically, the pCS6-*PRNP* plasmid was purchased from Transomic Technologies. The plasmid transfection process was performed with Lipofectamine 3000 according to the manufacturer's instructions with minimal modifications. The ADMSCs were replated 24 h prior to transfection at a density of 5 × 10^5^ cells in 4 ml of fresh culture medium in a 6-cm plastic dish. Since *PRNP* is the gene for PrP^C^, *PRNP* was replaced by PrP^C^ to facilitate an early understanding of this specific relationship in the present study.

### Cell grouping

**Figure 1:**
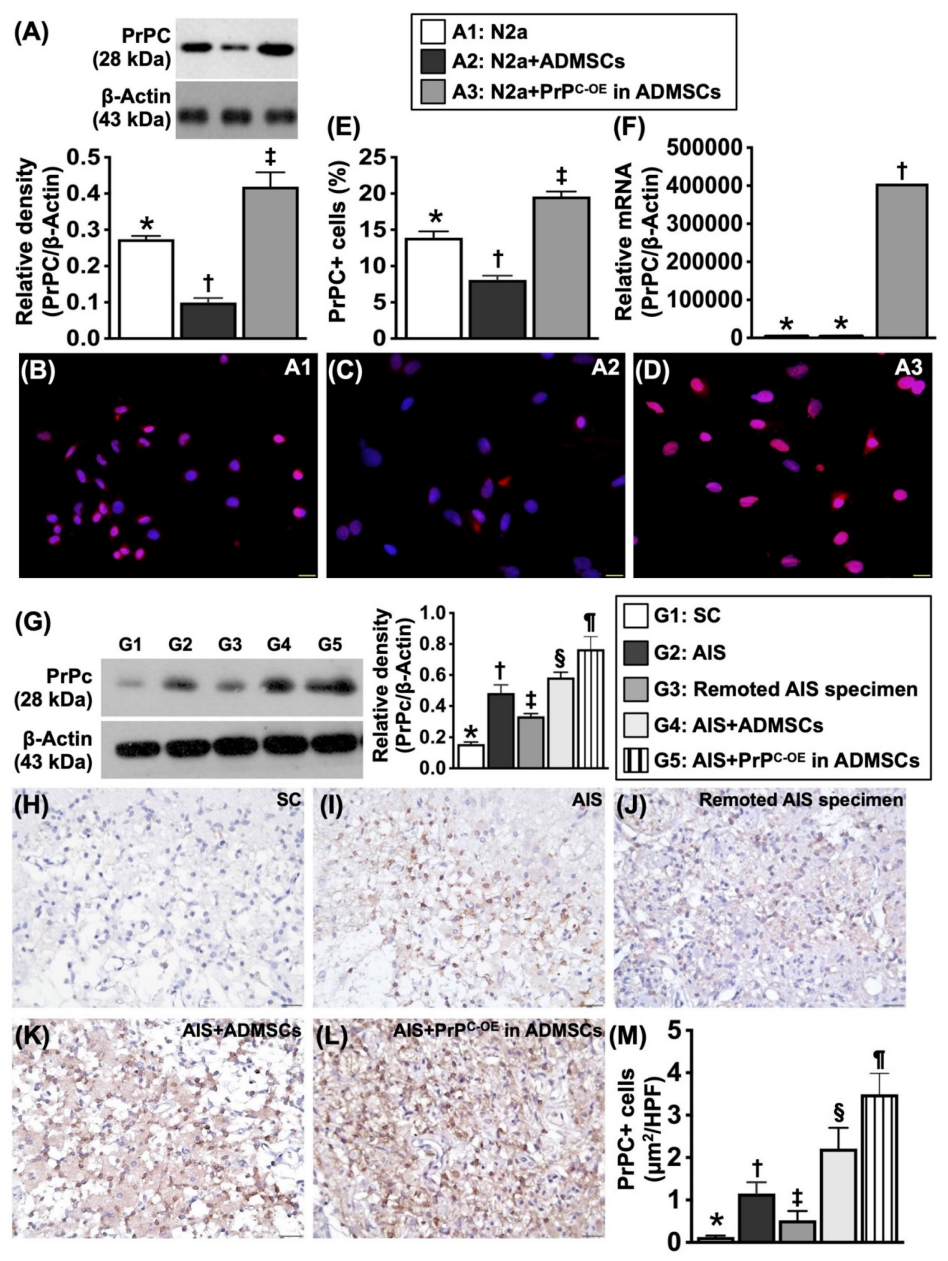
** Protein, cellular and gene expressions of cellular prion protein (PrP^C^) in N2a cell line and ADMSCs, and protein expression of PrP^C^ in different circumstances of brain tissues. A)** Protein expression of PrP^C^, * vs. other groups with different symbols (†, ‡), *P<*0.001. **B to D)** Illustrating immunofluorescent (IF) microscopic finding (400x) for delineating the cellular expression of PrP^C^ (pink color). Blue color indicated DAPI stain for identification of nuclei. **E)** Analytical result of PrP^C^ in cellular level, * vs. other groups with different symbols (†, ‡), *P<*0.001. Scale bar in right lower corner represents 20µm. **F)** Relative PrP^C^ mRNA level, * vs. ‡, *P<*0.001. All statistical analyses were performed by one-way ANOVA, followed by Bonferroni multiple comparison post hoc test (n=4 for each group). Symbols (*, †, ‡) indicate significance (at 0.05 level). A1 = N2a cells, A2 = adipose-derived mesenchymal stem cells (ADMSCs), A3 = cellular prion protein overexpression (PrP^C-OE^) in ADMSCs. **G)** Protein expression of PrP^C^ in different circumstances of harvested brain specimen, * vs. other groups with different symbols (†, ‡, §, ¶), *P<*0.0001. **H to L)** Illustrating the microscopic finding (400x) of IHC stain for identification of cellular expression of PrP^C^ (gray color). **M)** Analytical result of PrP^C^ in cellular level, * vs. other groups with different symbols (†, ‡, §, ¶), *P<*0.0001. Scale bar in right lower corner represents 20µm. Grouping: G1 = SC, G2 = acute ischemic stroke (AIS), G3 = remoted AIS specimen, G4 = AIS + ADMSCs, G5 = AIS + PrP^C-OE^ in ADMSCs. All statistical analyses were performed by one-way ANOVA, followed by Bonferroni multiple comparison post hoc test (n=3-5 for each group). Symbols (*, †, ‡) indicate significance (at 0.05 level). AIS = acute ischemic stroke; PrP^C-OE^ = cellular prion protein overexpression; ADMSCs = adipose-derived mesenchymal stem cells.

To test whether PrP^C^ expression was dissimilar in different cells, the cells were grouped into Groups A1 (N2a cells, i.e., the mouse neuroblastoma cell line), A2 (ADMSCs) and A3** (**ADMSCs with PrP^C-OE^). The results are shown in Figure 1.

**Figure 3:**
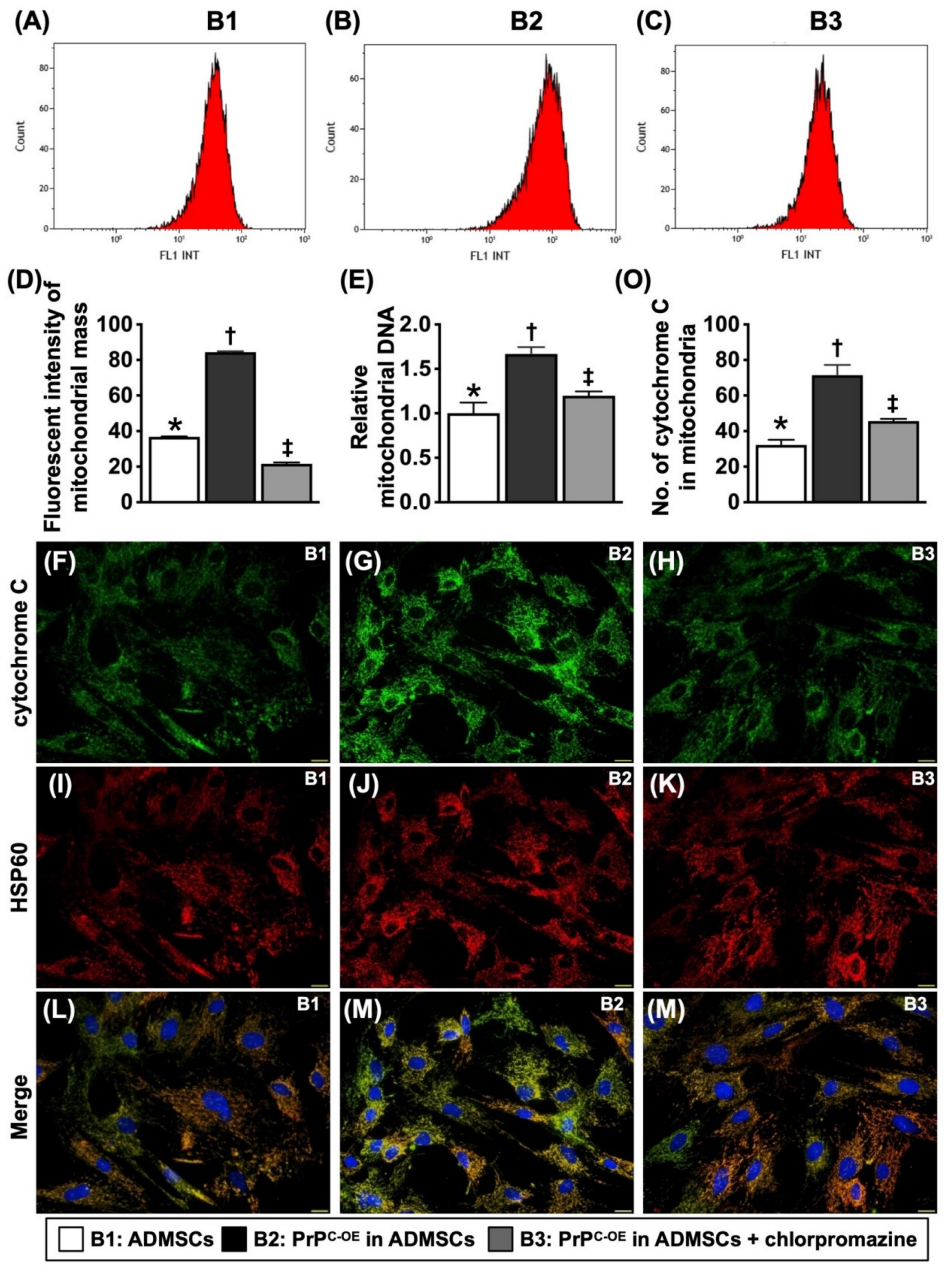
** The PrP^C^ augmented mitochondrial duplication in the cells. A to C)** Illustrating the flow cytometric analysis for determining the mitochondrial mass. **D)** Analytical result of mean fluorescent intensity of mitochondrial mass [Nonyl Acridine Orange (NAO), i.e., a green-fluorescent mitochondrial dye for measuring the *mitochondrial* content of cells], * vs. other groups with different symbols (†, ‡), *P<*0.0001. **E)** The relative mitochondrial DNA, * vs. other groups with different symbols (†, ‡), *P<*0.0001. **F to N)** Illustrating the IF microscopic finding (400x) for identification of cytochrome C (green color) (F to H), intracellular mitochondria (i.e., by HSP60 stain) (red color) (I to K) and the merged picture (i.e., combined cytochrome C and HSP60 staining) for identification of the expression of cytochrome C in mitochondria (green-pink color) (L to N). Scale bar in right lower corner represents 20µm. **O)** Analytical result of number of cytochrome C in mitochondria, * vs. other groups with different symbols (†, ‡), *P<*0.0001. All statistical analyses were performed by one-way ANOVA, followed by Bonferroni multiple comparison post hoc test (n=4-6 for each group). Symbols (*, †, ‡) indicate significance (at 0.05 level). B1 = ADMSCs, B2 = PrP^C-OE^ in ADMSCs, B3 = PrP^C-OE^ in ADMSCs + chlorpromazine. PrP^C-OE^ = cellular prion protein overexpression; ADMSCs = adipose-derived mesenchymal stem cells.

**Figure 4:**
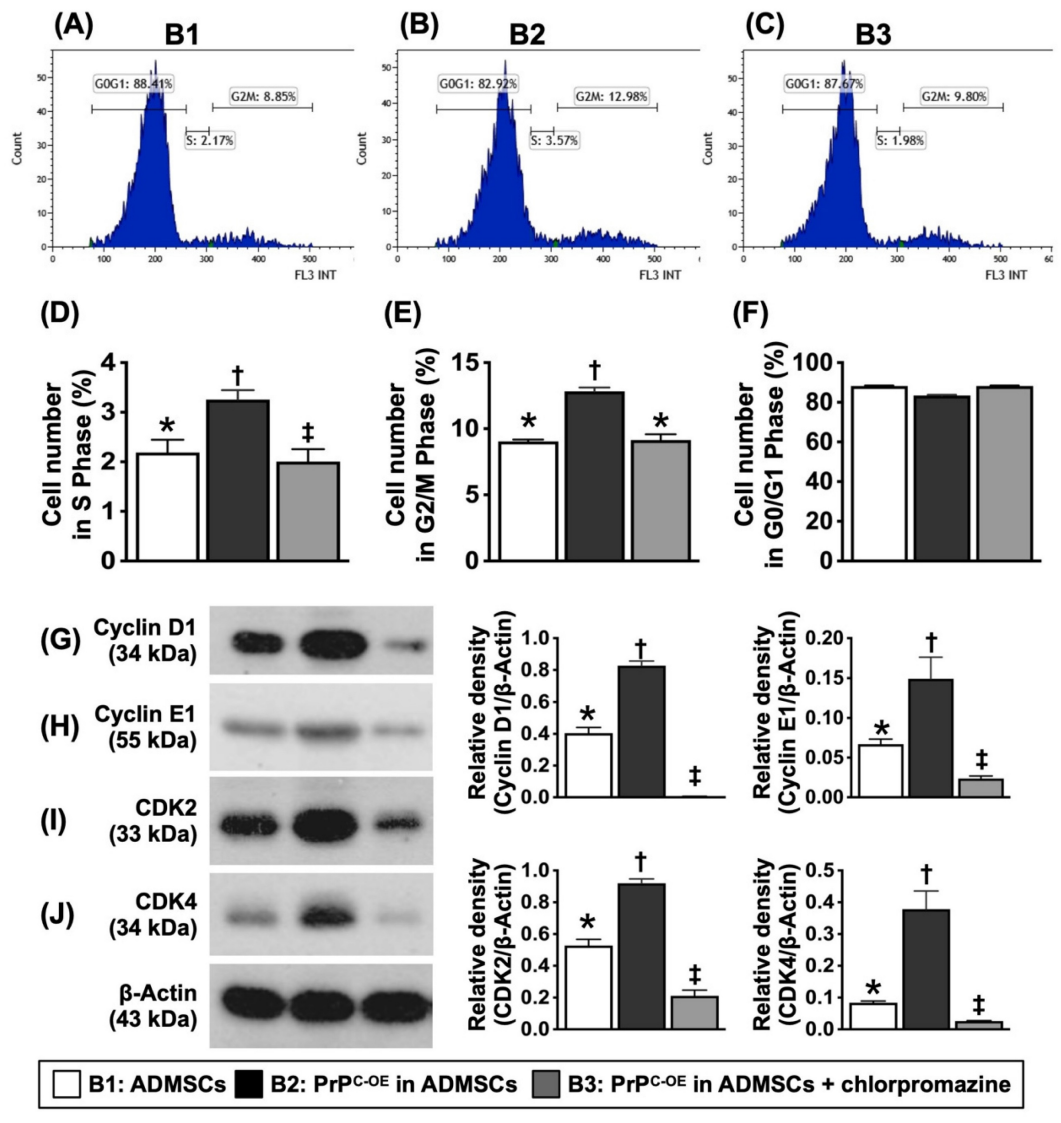
** The PrP^C^ upregulated synthetic/mitotic phases and augmented cell-cycle activation in the cells. A to C)** Illustrating the flow cytometric analysis for assessment of the mitotic and synthetic phases (i.e., cell cycle). **D)** Analytical result of synthesis phase (S), * vs. other groups with different symbols (†, ‡), *P<*0.0001. **E)** Analytical result of mitosis (G2/M) phase (i.e., mitotic cell cycle transition), * vs. other groups with different symbols (†, ‡), *P<*0.0001. **F)** Flow cytometric analysis of G0/G1 phase, p>0.5. **G)** Protein expression of Cyclin D1, * vs. other groups with different symbols (†, ‡), *P<*0.0001. **H)** Protein expression of Cyclin E1, * vs. other groups with different symbols (†, ‡), *P<*0.0001. **I)** Protein expression of CDK2, * vs. other groups with different symbols (†, ‡), *P<*0.0001. **J)** Protein expression of CDK4, * vs. other groups with different symbols (†, ‡), *P<*0.0001. All statistical analyses were performed by one-way ANOVA, followed by Bonferroni multiple comparison post hoc test (n=4-6 for each group). Symbols (*, †, ‡) indicate significance (at 0.05 level). B1 = ADMSCs, B2 = PrP^C-OE^ in ADMSCs, B3 = PrP^C-OE^ in ADMSCs + chlorpromazine treated for 24h, PrP^C-OE^ = cellular prion protein overexpression; ADMSCs = adipose-derived mesenchymal stem cells.

**Figure 5:**
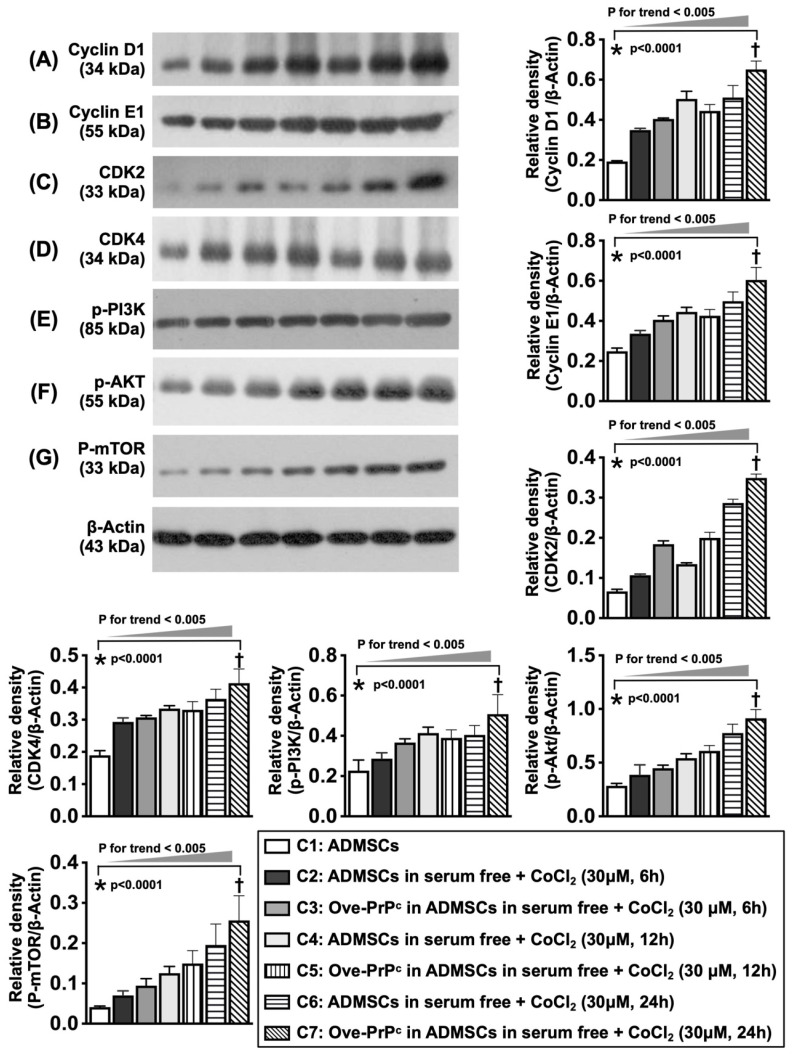
** Serial changes of cyclin D1, cell cycle subunits and cell stress signaling in condition of cellular prion protein overexpression and hypoxic condition. A)** Illustrating the protein expression of cyclin D1 at the time points of 0, 12 and 24 h, analytical results, * vs. †, *P<*0.001, *P* for trend <0.005. **B)** Illustrating the protein expression of cyclin E1 at time points of 0, 12 and 24 h, analytical result, * vs. †, *P<*0.001, *P* for trend <0.005. **C)** Illustrating the protein expression of cdk2 at time points of 0, 12 and 24 h, analytical result, * vs. †, *P<*0.001, *P* for trend <0.005. **D)** Illustrating the protein expression of cdk4 at time points of 0, 12 and 24 h, analytical result, * vs. †, *P<*0.001, *P* for trend <0.005. **E)** Illustrating the protein expression of phosphorylated (p)-PI3K at time points of 0, 12 and 24 h, * vs. †, *P<*0.001, *P* for trend <0.005. **F)** Illustrating the protein expression of P-Akt at time points of 0, 12 and 24 h, * vs. †, *P<*0.001, *P* for trend <0.005. **G)** Illustrating the protein expression of p-mTOR at time points of 6, 12 and 24 h, * vs. †, *P<*0.001, *P* for trend <0.005. C1 = ADMSCs, C2 = ADMSCs in serum free + CoCl_2_ (30 uM for 6 h), C3 = Ove-PrP^c^ in ADMSCs in serum free + CoCl_2_ (30 uM for 6 h), C4 = ADMSCs in serum free + CoCl_2_ (30 uM for 12 h), C5 = Ove-PrP^c^ in ADMSCs in serum free + CoCl_2_ (30 uM for 12 h), C6 = ADMSCs in serum free + CoCl_2_ (30 uM for 24 h), C7 = Ove-PrP^c^ in ADMSCs in serum free + CoCl_2_ (30 uM for 24 h). n = 3 for each group.

To test whether PrP^C^ participated in cell proliferation and augmented the wound healing rate, mitochondrial duplication and cell cycle activation, the cells were grouped into Groups B1 (ADMSCs), B2 (ADMSCs with PrP^C-OE^), and B3 [ADMSCs with PrP^C-OE^ + promazine (i.e., an inhibitor of PrP^C^ formation)]. The results are shown in Figures 3 to 5.

**Figure 6:**
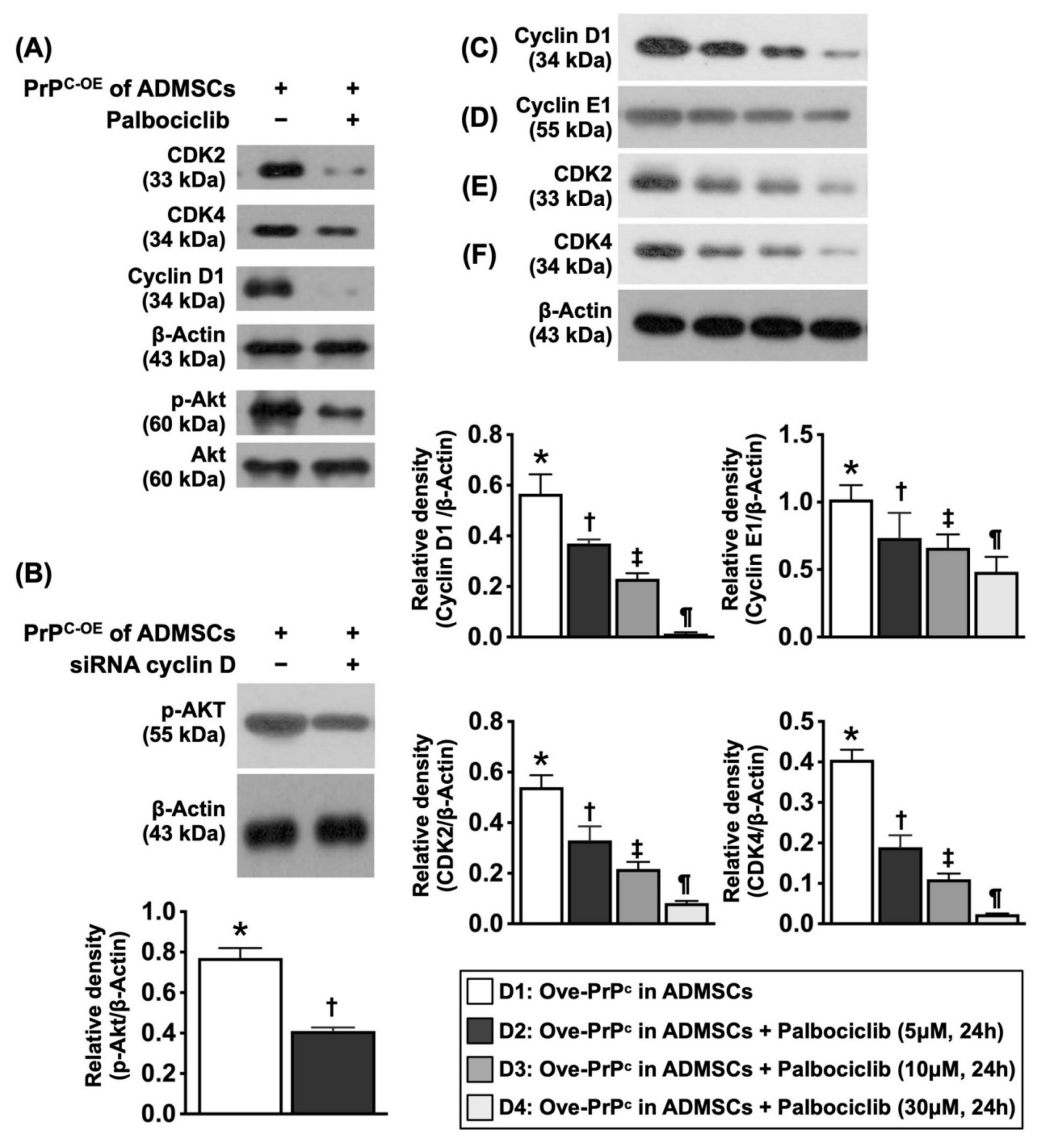
** The cardinal enzymes of cyclin D1/CDK on regulating cell cycle subunits and the PrP^C^-mediated cell stress/proliferation signaling in inhibited condition of CDK. A)** Illustrating the protein expressions of CDK2, CDK4, cyclin D1 and phosphorylated (p) were notably reduced after palbociclib (30 µM) treatment, implicating that inhibited CDK affected the expression of cyclin D1 and p-Akt (refer to Figure 10F). **B)** Protein expression of p-Akt was markedly reduced in siRNA (i.e., silencing) cyclin D in PrP^C-OE^ of ADMSCs than in that of PrP^C-OE^ of ADMSCs only, * vs. †, *P<*0.001. **C)** Protein expression of cyclin D1, * vs. other groups with different symbols (†, ‡, §), *P<*0.001. **D)** Protein expression of cyclin E1, * vs. other groups with different symbols (†, ‡, §), *P<*0.001. **E)** Protein expression of ckd2, * vs. other groups with different symbols (†, ‡, §), *P<*0.001. **F)** Protein expression of ckd4, * vs. other groups with different symbols (†, ‡, §), *P<*0.001. All statistical analyses were performed by one-way ANOVA, followed by Bonferroni multiple comparison post hoc test (n=3 for each group). Symbols (*, †, ‡, §) indicate significance (at 0.05 level). D1 = PrP^C-OE^ in ADMSCs, D2 = PrP^C-OE^ in ADMSCs + Palbociclib (5 uM for 24h), D3 = PrP^C-OE^ in ADMSCs + Palbociclib (10 uM for 24h), D4 = PrP^C-OE^ in ADMSCs + Palbociclib (30 uM for 24h). Palbociclib = a specific inhibitor of cyclin-dependent protein kinase.

To elucidate whether the protein levels of cyclin D1/CDK were consistent with the upregulation of cell stress signaling, the cell cultures were grouped into Groups C1 (ADMSCs), C2 [ADMSCs in serum-free medium + CoCl_2_ (30 µM for 6 h)], C3 [ADMSCs with PrP^C-OE^ in serum-free medium + CoCl_2_ (30 µM for 6 h)], C4 [ADMSCs in serum-free medium + CoCl_2_ (30 µM for 12 h)], C5 [ADMSCs with PrP^C-OE^ in serum-free medium + CoCl_2_ (30 µM for 12 h)], C6 [ADMSCs in serum-free medium + CoCl_2_ (30 µM for 24 h)] and C7 [ADMSCs with PrP^C-OE^ in serum-free medium + CoCl_2_ (30 µM for 24 h)]. The results are shown in Figure 6. The dosage of CoCl_2_ used in the present study was based on our previous report 38 with some modifications.

To elucidate whether cyclin D1/CDK were the crucial regulatory enzymes involved in the upregulation of cell proliferation signaling through the regulation of cell cycle checkpoints (i.e., PI3K/Akt), stepwise concentrations (0, 5, 10, and 30 µM) of palbociclib (i.e., an inhibitor of cyclin D1/CDK) were utilized, and the cell cultures were grouped into Groups D1 (ADMSCs with PrP^C-OE^), D2 [ADMSCs with PrP^C-OE^ + palbociclib (5 µM for 24 h)], D3 [ADMSCs with PrP^C-OE^ + palbociclib (10 µM for 24 h)] and D4 [ADMSCs with PrP^C-OE^ + palbociclib (30 µM for 24 h)].

### Animal model of acute IS and animal grouping

The protocol and procedure were based on our previous study 27. In detail, adult male Sprague‒Dawley rats weighing 350-375 g (Charles River Technology, BioLASCO Taiwan Co., Ltd., Taiwan) were used in this study. All animals were anesthetized with 2% inhalational isoflurane in a supine position on a warming pad (37 °C). After the left common carotid artery (LCCA) was exposed through a transverse neck incision, a small arteriotomy was performed on the LCCA, through which a 0.28 mm diameter nylon monofilament was carefully pushed forward into the distal LCCA for occlusion of the left middle cerebral artery to induce brain ischemia and infarction of its supplied region. The nylon monofilament was removed 60 min after occlusion, followed by closure of the muscle and skin in layers. The duration of nylon monofilament occlusion to the left middle cerebral artery was based on our previous study 27. The rats were then cared for in a portable animal intensive care unit (ThermoCare**^®^**) with food and water for 24 h. For those animals in the sham-operated control (SC) group, only the skin and muscle layers were opened, and these layers were immediately closed after the LCCA was identified.

After acute IS induction, the animals were then categorized into Group 1 (SC, i.e., incision of the neck skin and dissection of the LCCA only), Group 2 (acute IS only), Group 3 (acute IS + ADMSCs (1.2 × 10^6^) each time at 1.5 and 24 h after IS) and Group 4 (acute IS + ADMSCs with PrP^C-OE^ (1.2 × 10^6^) each time at 1.5 and 24 h after IS). The dose of ADMSCs used in the present IS animal model was based on our previous investigations 28-32.

### Corner test for the evaluation of neurological function

The sensorimotor functional test (corner test) was conducted for each rat at baseline and at 3, 7, 14 and 28 days after acute IS induction, as we previously described 1, 2. Briefly, the rats in each group were allowed to walk through a tunnel and then into a corner, the angle of which was 60 degrees. To exit the corner, the rat could turn either left or right. A technician who was blinded to the study design recorded the results. This test was repeated 10 to 15 times, with at least 30 s between each test. The technician recorded the number of right and left turns from 10 successful trials for each animal, and the results were used for statistical analysis.

### Western blot analysis

The procedure and protocol for Western blot analysis were based on our recent reports 27, 31, 36. Briefly, equal amounts (50 μg) of protein extracts were loaded and separated by SDS‒PAGE with acrylamide gradients. After electrophoresis, the separated proteins were transferred electrophoretically to a polyvinylidene difluoride membrane (GE, UK). Nonspecific sites were blocked by incubation of the membrane in blocking buffer [5% nonfat dry milk in T-TBS (TBS containing 0.05% Tween 20)] overnight. The membranes were incubated with the indicated primary antibodies [anti-PrP^C^ (1:1000, Abcam), anti-Bax (1:1000, Abcam), anti-cleaved caspase 3 (1:1000, Cell Signaling), anti-cleaved PARP (1:1000, Cell Signaling), anti-beclin-1 (1:1000, Cell Signaling), anti-Atg5 (1:1000, Cell Signaling), anti-phosphorylated (p)-DRP1 (1:1000, Cell Signaling), anti-DRP1 (1:1000, Abcam), anti-cyclophilin D (1:10000, Abcam), anti-cytochrome C (1:10000, BD), p-γ-H2AX (1:1000, Cell Signaling), anti-tumor necrosis factor (TNF)-α (1:1000, Cell Signaling), anti-matrix metalloproteinase (MMP)-9 (1:1000, Cell Signaling), anti-LC3B-I (1: 1000, Cell Signaling), anti-LC3B-II (1: 1000, Cell Signaling) and anti-actin (1: 1000, Millipore)] for 1 hour at room temperature. Horseradish peroxidase-conjugated anti-rabbit immunoglobulin IgG (1:2000, Cell Signaling, Danvers, MA, USA) was used as a secondary antibody for a one-hour incubation at room temperature. The washing procedure was repeated eight times within one hour. Immunoreactive bands were visualized by enhanced chemiluminescence (ECL; Amersham Biosciences, Amersham, UK) and exposed to Biomax L film (Kodak, Rochester, NY, USA). For the purpose of quantification, the ECL signals were digitized by LabWorks software (UVP, Waltham, MA, USA).

### Immunofluorescent (IF) and immunohistochemical (IHC) staining of brain specimens

The procedures and protocols for IF and IHC staining were based on our previous reports 27, 31, 36. In detail, frozen sections (4 μm thick) were obtained from the brain hemorrhagic area/at risk area of each animal, permeabilized with 0.5% Triton X-100, and incubated with antibodies against NeuN (1:100, Merck), CD31 (1:100, Abcam), von Willebrand factor (vWF) (1:200, Abcam), CXCR4 (1:100, Invitrogen), CD14 (1:200, Proteintech), and F4/80 (1:100, Santa Cruz) at 4 °C overnight. Alexa Fluor 488, Alexa Fluor 568, or Alexa Fluor 594-conjugated goat anti-mouse or rabbit IgG was used to localize the signals. The sections were finally counterstained with DAPI and observed with a fluorescence microscope equipped for epifluorescence microscopy (Olympus IX-40). Three brain sections from each rat were analyzed. For quantification, three randomly selected high-power fields (HPFs; 400x for IF study) were analyzed in each section. The mean number of positively stained cells per HPF for each animal was then determined by adding all the numbers and then dividing by 9.

### Statistical analysis

The quantitative data are expressed as the means ± SDs. Statistical analysis was performed with ANOVA, followed by the Bonferroni multiple-comparison post hoc test. SAS statistical software for Windows version 8.2 (SAS Institute, Cary, NC, USA) was utilized. A p value of less than 0.05 was considered statistically significant.

## Results

### Assessment of the protein and gene expression of the cellular prion protein (PrP^C^) in the N2a cell line, MSCs and brain tissues (Figure 1)

To test whether PrP^C^ expression was dissimilar in different cells, Western blot and gene analyses and IHC staining were utilized. The cells were grouped into groups A1 (N2a cells), A2 (ADMSCs) and A3 (ADMSCs with PrP^C-OE^). The results revealed that the protein expression levels of PrP^C^ were significantly greater in A3 than in A1 and A2 and significantly greater in A1 than in A2. Additionally, the mRNA expression of PrP^C^ was also significantly greater in A3 than in A1 and A2.

To test whether PrP^C^ expression is upregulated in brain tissue after acute IS, WB and IHC staining were conducted. The results demonstrated that the protein expression level of PrP^C^ was highest in the acute IS + ADMSCs with PrP^C-OE^ group, lowest in the SC group, significantly lower in remote brain tissue of the acute IS group than in that of the acute IS and acute IS + ADMSCs groups and significantly lower in the acute IS group than in the acute IS + ADMSCs group, indicating that ischemic stroke stimulated PrP^C^ expression and that treatment with ADMSCs with PrP^C-OE^ further enhanced the expression of this protein in brain tissue.

### PrP^C^ increased cell viability, cell proliferation and the wound healing rate (Figure 2)

To test whether PrP^C^ participated in cell proliferation and augmented the wound healing rate, three in vitro cell culture groups, B1 (ADMSCs), B2 (ADMSCs with PrP^C-OE^) and B3 [ADMSCs with PrP^C-OE^ + chlorpromazine (i.e., an inhibitor of PrP^C^ formation)], were used.

The results of the MTT assay revealed that cell viability was significantly greater in B1 and significantly greater in B2 than in B3, regardless of the time point (24, 48 or 72 h). Additionally, at 72 h, the cellular expression of PCNA and BrdU uptake, two indicators of cell proliferation, displayed an identical pattern in the MTT assay among the groups. Furthermore, at 24 h, the wound healing rate also exhibited a pattern identical to that of the MTT assay among the groups. These findings suggested that PrP^C^ played a crucial role in cell growth and proliferation.

### PrP^C^ augmented mitochondrial duplication in the cells (Figure 3)

To determine whether the number of intracellular mitochondria consistently increases with increasing cell viability and proliferation, the cell grouping as shown in Figure 3 was utilized. By 48 h, flow cytometric and qPCR analyses revealed that the mitochondrial mass and relative amount of mitochondrial DNA were significantly greater in B2 than in B1, and these changes were significantly reversed in B3. Additionally, IF staining analysis revealed that the number of mitochondrial cytochrome C molecules, an indicator of mitochondrial integrity, displayed a similar pattern to that of the mitochondrial mass among the groups.

### PrP^C^ augmented the synthetic and mitotic phases and cell cycle activation in ADMSCs (Figure 4)

Flow cytometric analysis demonstrated that the number of cells in the synthetic and mitotic phases was significantly greater in B2 than in B1. However, these two parameters were significantly reversed in B3. Additionally, the protein levels of cyclin D1, cyclin E1, CDK2, and CDK4, four indices of the cell cycle, exhibited an identical pattern to that of the mitochondrial mass among the groups, indicating that PrP^C-OE^ accelerated the activation of the cell cycle and the synthetic and mitotic phases.

### Hypoxia was involved in changes in cyclin D1, cell cycle subunits and cell stress signaling (Figure 5)

To elucidate whether the protein levels of cyclin D1/cell cycle subunits are consistent with the upregulation of cell stress signaling, the cell culture Groups C1 to C7 were analyzed. Compared with those at baseline, the protein levels of D1, an indicator of cell proliferation that regulates cell cycle checkpoints and transcriptional events in response to extracellular and intracellular signals, cell cycle subunits (cyclin E1, CDK2, and CDK4), and cell stress/proliferation signaling proteins (i.e., p-PI3K, p-Akt, and p-mTOR) were significantly upregulated at 6, 12 and 24 h after 6 h of hypoxic stimulation.

### Cyclin D1/CDK were the primary enzymes regulating the PrP^C^-mediated cell stress and proliferation signaling (Figure 6)

To elucidate whether cyclin D1/CDK are the crucial regulatory enzymes involved in the upregulation of the cell cycle checkpoints and the checkpoint of PrP^C-OE^-mediated cell proliferation signaling (i.e., PI3K/Akt), as well as in the transcriptional events in response to extracellular and intracellular stimulation, palbociclib (PD0332991) (30 µM), siRNA silencing of cyclin D1, and stepwise increasing concentrations of palbociclib (PD0332991) (i.e., 0, 5, 10, and 30 µM) were utilized in the culture of ADMSCs with PrP^C-OE^.

The results demonstrated that the protein expression levels of cyclin-dependent kinase 2 (CDK2) and CDK4 were substantially lower in ADMSCs with PrP^C-OE^ than in those treated with palbociclib (30 µM). Additionally, the protein expression levels of cyclin D1 and p-Akt presented an identical pattern to that of CDK2/CDK4 within the two groups. Additionally, the protein expression of p-Akt in ADMSCs with siRNA cyclin D1 and PrP^C-OE^ was markedly lower than that in ADMSCs with PrP^C-OE^ only.

Furthermore, the protein expression levels of cyclin D1, cyclin E1, CDK2, CDK4 and p-Akt significantly and progressively decreased as the concentration of palbociclib increased. These findings highlighted that cyclin D1/CDK was the important checkpoint of PrP^C-OE^-mediated cell proliferation (refer to Figure 10).

### Time courses of neurological function and inflammatory and fibrotic parameters in ischemic brain tissues by Day 28 after acute IS (Figure 7)

To evaluate whether ADMSCs with and without PrP^C-OE^ improve neurological function, a corner test was conducted on Day 0 prior to acute IS induction and at Days 3, 7, 14 and 28 after acute IS induction. The results demonstrated that, on Day 0, neurological function did not differ among the groups. However, on Day 3 after acute IS induction, neurological function was significantly impaired in Group 2 (acute IS only), Group 3 (acute IS + ADMSCs) and Group 4 (acute IS + ADMSCs with PrP^C-OE^) compared with that in Group 1 (SC), but neurological function did not differ among Groups 2 to 4. On the other hand, by Days 7, 14 and 28 after acute IS induction, this parameter was still impaired in Group 2 compared with that in Group 1 but was significantly reversed in Group 3 and further significantly reversed in Group 4.

**Figure 8:**
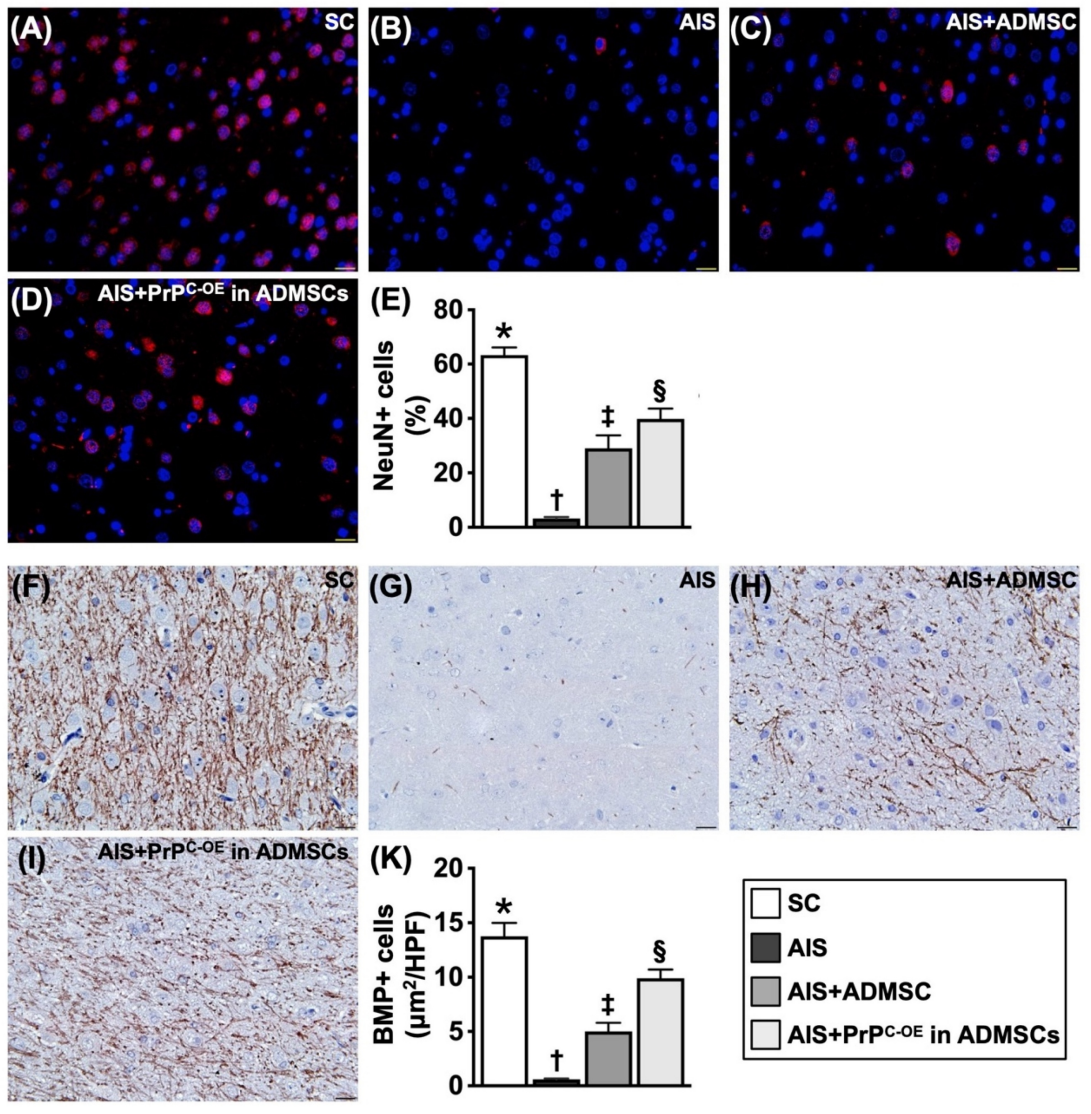
** ADMSCs/PrP^C-OE^ in ADMSCs treatment effectively preserved the microstructural integrity of the neurons and myelin sheath by day 28 after acute IS. A to D)** Illustrating the IF microscopic finding (400x) for identification of NeuN+ cells (pink color). **E)** Analytical result of number of NeuN+ cells, * vs. other groups with different symbols (†, ‡, §), *P<*0.0001. **F to I)** Illustrating the IHC microscopic finding (400x) for identification of cellular expression of myelin basic protein (*MBP*) (gray color). **J)** Analytical result of cellular expression of positively stained *MBP,* * vs. other groups with different symbols (†, ‡, §), *P<*0.0001. Scale bar in right lower corner represents 20µm. All statistical analyses were performed by one-way ANOVA, followed by Bonferroni multiple comparison post hoc test (n=6 for each group). Symbols (*, †, ‡, §) indicate significance (at 0.05 level). SC = sham-operated control; IS = ischemic stroke; PrP^C-OE^ in ADMSCs = cellular prion protein overexpression in adipose-derived mesenchymal stem cells (ADMSCs).

To investigate whether ADMSCs with and without PrP^C-OE^ repress the inflammatory reaction and fibrotic formation, we utilized IF and IHC staining in an in vivo study. The results revealed that the cellular expression level of CD68, an index of inflammation, and the fibrotic area (i.e., by Masson's trichrome stain) were lowest in Group 1, highest in Group 2, and significantly greater in Group 3 than in Group 4. Furthermore, the protein levels of TNF-α and MMP-9, two inflammatory biomarkers, were also expressed at similar levels to that of CD68. The findings in Figure 8, therefore, suggested that ADMSCs with PrP^C-OE^ were superior to ADMSCs without PrP^C-OE^ in suppressing inflammation and fibrosis and improving neurological outcomes.

### Treatment with ADMSCs with and without PrP^C-OE^ preserved the microstructural integrity of neurons and the myelin sheath by Day 28 after acute IS (Figure 8)

IHC staining findings revealed that the cellular expression of myelin basic protein (*MBP*), an indicator of myelin sheath integrity, was highest in Group 1, lowest in Group 2 and significantly lower in Group 3 than in Group 4. Additionally, the IF staining findings demonstrated that the cellular expression level of NeuN, an marker of the integrity of neurons in the brain, exhibited a similar pattern to that of MBP among the groups.

### Assessment of the protein expression levels of markers of apoptosis, mitochondrial and DNA damage and autophagy in ischemic brain tissues at Day 28 after acute IS (Figure 9)

The protein expression levels of Bax, cleaved caspase 3 and cleaved PARP, three indicators of apoptosis, were significantly greater in Group 2 than in Group 1; these changes were reversed in Group 3 and further reversed in Group 4. Additionally, the protein expression levels of beclin-1 and Atg5 and the ratio of LC3BII to LC3BI, three indicators of autophagy, displayed an identical pattern to those of the apoptosis markers among the groups. Furthermore, the protein expression levels of cytosolic cytochrome C, p-DRP1 and cyclophilin D, three indices of mitochondrial damage, and the protein expression level of γ-H2AX, an index of DNA damage, exhibited identical patterns to those of the apoptosis markers among the groups.

### Brain ischemic volume (BIV) by Day 28 after acute IS and schematic illustration of the underlying mechanism (Figure 10)

To further evaluate the anatomical features of the brain parenchyma, we utilized a brain MRI instrument. As we expected, by Day 28 after acute IS induction, brain MRI revealed that the BIV was lowest in Group 1, highest in Group 2 and significantly lower in Group 4 than in Group 3.

On the basis of our extensive works in the present study, we schematically illustrated the proposed mechanism by which the coordination between cyclin D1/CDK and PrP^C^ activates mitogenic/cell proliferation signaling to improve neurological outcomes in IS rodents.

## Discussion

This study investigated the underlying mechanism of the coordination between cyclin D1/CDK and PrP^C^ to activate cell stress/cell proliferation and cell cycle signaling during treatment with ADMSCs with and without PrP^C-OE^ to protect neurological function and neurological microstructural integrity in rats after acute IS, which has several notable implications. First, compared with that in the SC animals, the protein level of PrP^C^ was significantly increased in the remote areas of the IS and in the IS area with and without ADMSC treatment and further increased in the IS area treated with ADMSCs with PrP^C-OE^. Second, the results of the in vitro study demonstrated that PrP^C^ played a crucial role in increasing cell viability, proliferation and the wound healing rate and further upregulated the mitotic phases and cell cycle activation after treatment with ADMSCs with PrP^C-OE^. Third, the results of the in vitro study revealed that cyclin D1/CDK was the checkpoint regulating PrP^C^-mediated cell stress/proliferation signaling and promoting the rate of the cell cycle.

Intriguingly, our previous study demonstrated that PrP^C^, which was markedly increased in the myocardium after ischemia‒reperfusion injury, was essential for myocardial regeneration 39. An essential finding in the present study was that, compared with those in the control group, the gene and protein expression levels of PrP^C^ were markedly increased in the IS brain tissue and remote areas of the IS brain tissue, increased in the ADMSCs-treated brain tissues and presented further increases in the PrP^C-OE^ ADMSCs-treated brain tissue. These findings highlighted that PrP^C^ expression in brain tissues is commonly increased even in remote brain areas in response to ischemic stimulation and further increased in IS tissue after receiving combined IS stimulation and treatment with PrP^C-OE^ ADMSCs. Accordingly, these findings strengthened the findings of our previous study 39.

The principal findings of the present study were that PrP^C^ plays a fundamental role in increasing cell viability, cell stress/cell proliferation signaling, the wound healing process, and the generation of mitochondria, as well as promoting the rate of cell cycle and mitogenic signaling. Interestingly, our recent study 40 revealed that the rejuvenation of endothelial progenitor cells by PrP^C-OE^ alleviated critical limb ischemia in rats and restored blood flow in the ischemic area, mainly through augmenting angiogenesis and cell stress signaling (i.e., p-PI3K/p-Akt/p-m-TOR). Additionally, our other recent study 41 revealed that valsartan/melatonin-facilitated ADMSCs safeguard residual kidney function in chronic kidney disease rats, mainly through increasing PrP^C^ levels, resulting in the activation of cell stress signaling. Furthermore, our more recent study 36 revealed that ADMSCs with PrP^C-OE^ effectively protected the rodent kidney against ischemia‒reperfusion injury, principally through increasing ATP/mitochondrial biogenesis. Although the results of our recent reports 36, 39, 41 corroborated the findings of the present study, the exact mechanism by which PrP^C^/PrP^C-OE^ in ADMSCs contributes to protecting organs against ischemia-induced damage and dysfunction has not been clearly delineated by our recent investigations 36, 40, 41.

The most important finding in the present study was that cyclin D1/CDK coordinated with PrP^C^ to promote cell stress/cell proliferation and cell cycle signaling, especially when PrP^C-OE^ in ADMSCs was considered strategic management. Our findings clearly explained why the markers of inflammation, fibrosis, apoptosis, mitochondrial and DNA damage and autophagy were markedly reduced, whereas neurological function and neurological microstructural integrity were markedly increased in rat IS after receiving treatment with ADMSCs with and without PrP^C-OE^. We clearly and comprehensively show schematically **(Figure 10F)** the underlying mechanism by which neurological outcomes are significantly improved in the IS animals treated with ADMSCs with and without PrP^C-OE^.

### Study limitations

**Figure 9:**
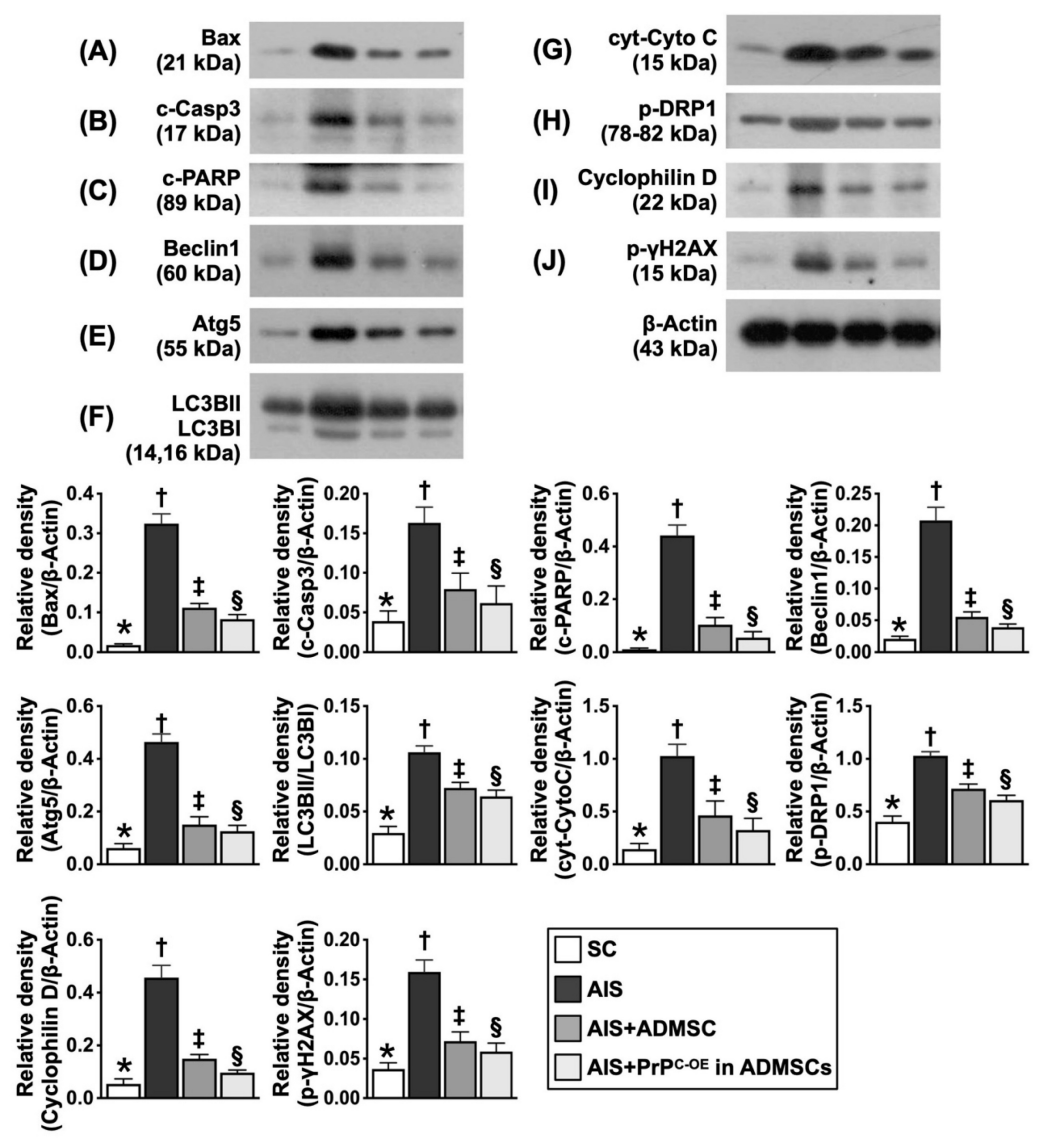
**Protein expressions of apoptosis, mitochondrial/DNA damage and autophagy. A)** Protein expression of Bax, * vs. other groups with different symbols (†, ‡, §), *P<*0.0001. **B)** Protein expression of cleaved caspase 3 (c-Casp3), * vs. other groups with different symbols (†, ‡, §), *P<*0.0001. **C)** Protein expression-PARP, * vs. other groups with different symbols (†, ‡, §), *P<*0.0001. **D)** Protein expression of beclin-1, * vs. other groups with different symbols (†, ‡, §), *P<*0.0001. **E)** Protein expression of Atg5, * vs. other groups with different symbols (†, ‡, §), *P<*0.0001. **F)** Protein expression of the ratio of LC3BII to LC3BI, * vs. other groups with different symbols (†, ‡, §), *P<*0.0001. **G)** Protein expressions of cytosolic cytochrome C (cyt-CytoC), * vs. other groups with different symbols (†, ‡, §), *P<*0.0001. **H)** Protein expression of phosphorylated (p)-dynamin-1-like protein (DPR1), * vs. other groups with different symbols (†, ‡, §), *P<*0.0001. **I)** Protein expression of cyclophilin D, * vs. other groups with different symbols (†, ‡, §), *P<*0.0001. **J)** Protein expression of γ-H2AX, * vs. other groups with different symbols (†, ‡, §), *P<*0.0001. All statistical analyses were performed by one-way ANOVA, followed by Bonferroni multiple comparison post hoc test (n=6 for each group). Symbols (*, †, ‡, §) indicate significance (at 0.05 level). SC = sham-operated control; IS = ischemic stroke; PrP^C-OE^ in ADMSCs = cellular prion protein overexpression in adipose-derived mesenchymal stem cells (ADMSCs).

**Figure 10:**
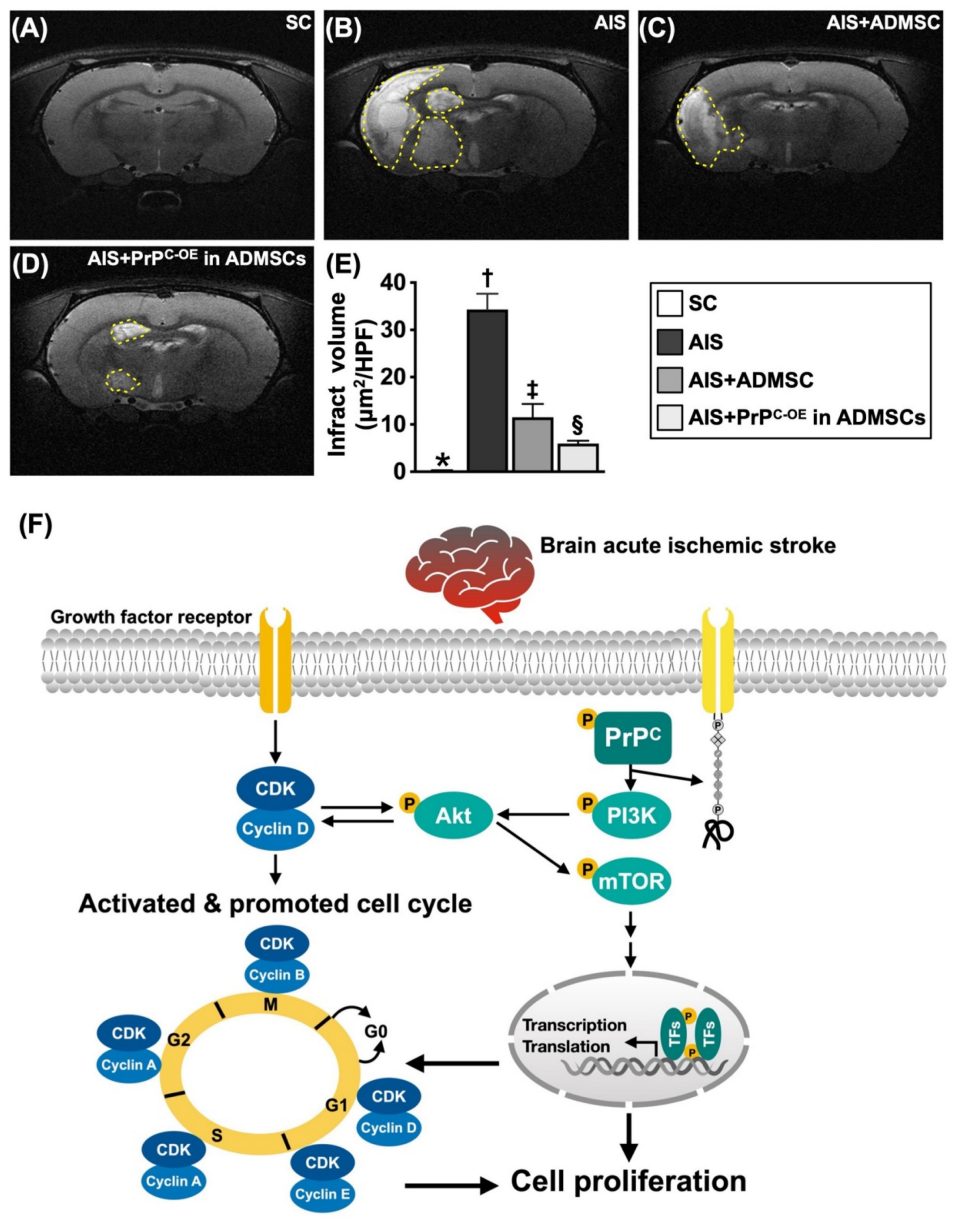
** Brain ischemic volume (BIV) by day 28 after acute IS induction and proposed underlying mechanism. A to D)** Illustrating the brain magnetic resonance imaging (MRI) finding for identification of BIV (white color) (yellow dotted-line area). **E)** Analytical result of BIV (i.e., the percentage of the whole brain volume), * vs. other groups with different symbols (†, ‡, §), *P<*0.0001. **F)** Based on our extensive works in the present study, we schematically illustrated the proposed mechanism of coordination of cyclin D1/CDK and PrP^C^ on activating mitogenic/cell-proliferation signaling for improving neurological outcomes in AIS rodent. CDK = cyclin-dependent kinase; PrP^C^ = cellular prion protein.

This study has several limitations. First, the study period of only 28 days was relatively short, and long-term outcomes after MSCs treatment are currently unknown. Second, without the use of conditional gene knockout to eliminate gene effects in the brain, there is no direct evidence that the coordination of PrP^C^ and cyclin D1/CDK in the brain can improve neurological outcomes after AIS. Third, to detect changes in autophagy accurately, multiple techniques need to be used simultaneously, such as the use of inhibitors of different stages of autophagy, electron microscopy, and a double-label mCherry-GFP-LC3B adenovirus, to produce convincing experimental results. However, the parameters of autophagy shown in Figure 9 were only assessed by Western blot analysis. Additionally, the LC3-II level can be easily affected by the duration of the experiment due to the rapid autophagy process, which can result in the possibility of inaccuracy. Accordingly, we would like to caution the readers to interpret the parameters judiciously.

In conclusion, the results of the present study demonstrated that the coordination of cyclin D1/CDK with PrP^C^ in ADMSCs activated cell stress/proliferation signaling and promoted the rate of the cell cycle, resulting in improved neurological outcomes in rodents after acute IS.

## Figures and Tables

**Figure 2:**
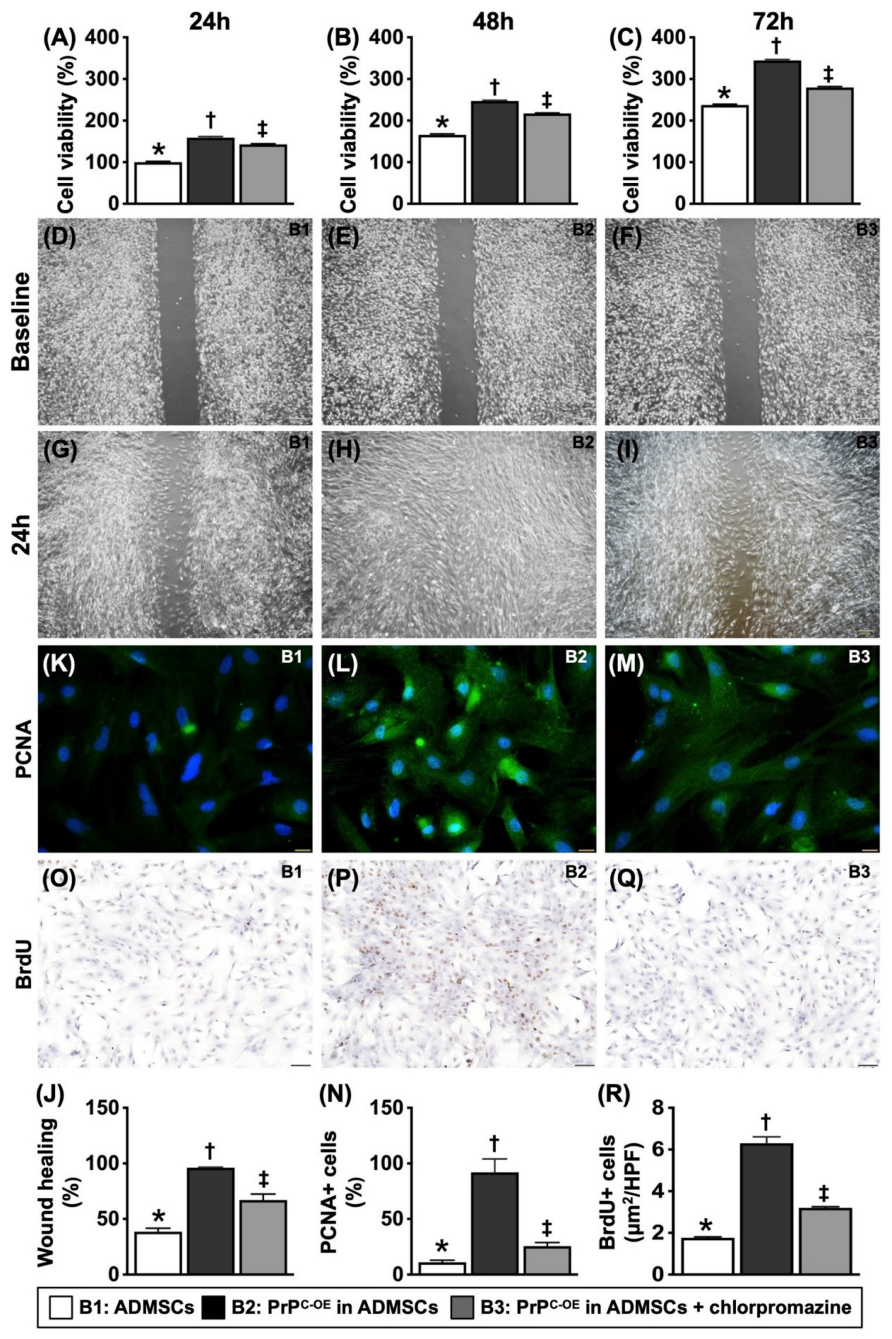
**Crucial role of PrP^C^ on cell growth and proliferation. A)** Cell viability at 24h, * vs. other groups with different symbols (†, ‡), *P<*0.0001. **B)** Cell viability at 48h, * vs. other groups with different symbols (†, ‡), *P<*0.0001. **C)** Cell viability at 72h, * vs. other groups with different symbols (†, ‡), *P<*0.0001. **D to F)** Illustrating the wound healing process at baseline (i.e., 0h), *P*>0.5. **G to I)** Illustrating the wound healing process at 24h. **J)** Analytical result of wound healing rate, * vs. other groups with different symbols (†, ‡), *P<*0.0001. **K to M)** Illustrating the immunofluorescent (IF) microscopic finding (400x) for identification of cellular expression of PCNA (green color). Scale bar in right lower corner represents 20µm. **N)** Analytical result of number of PCNA+ cells, * vs. other groups with different symbols (†, ‡), *P<*0.0001. **O to Q)** Illustrating the microscopic finding (100x) for identification of BrdU+ cells (gray color). Scale bar in right lower corner represents 100µm. **R)** Analytical result of number of BrdU+ cells, * vs. other groups with different symbols (†, ‡), *P<*0.0001. All statistical analyses were performed by one-way ANOVA, followed by Bonferroni multiple comparison post hoc test (n=6 for each group). Symbols (*, †, ‡) indicate significance (at 0.05 level). B1 = ADMSCs, B2 = PrP^C-OE^ in ADMSCs, B3 = PrP^C-OE^ in ADMSCs + chlorpromazine. PrP^C-OE^ = cellular prion protein overexpression; ADMSCs = adipose-derived mesenchymal stem cells.

**Figure 7:**
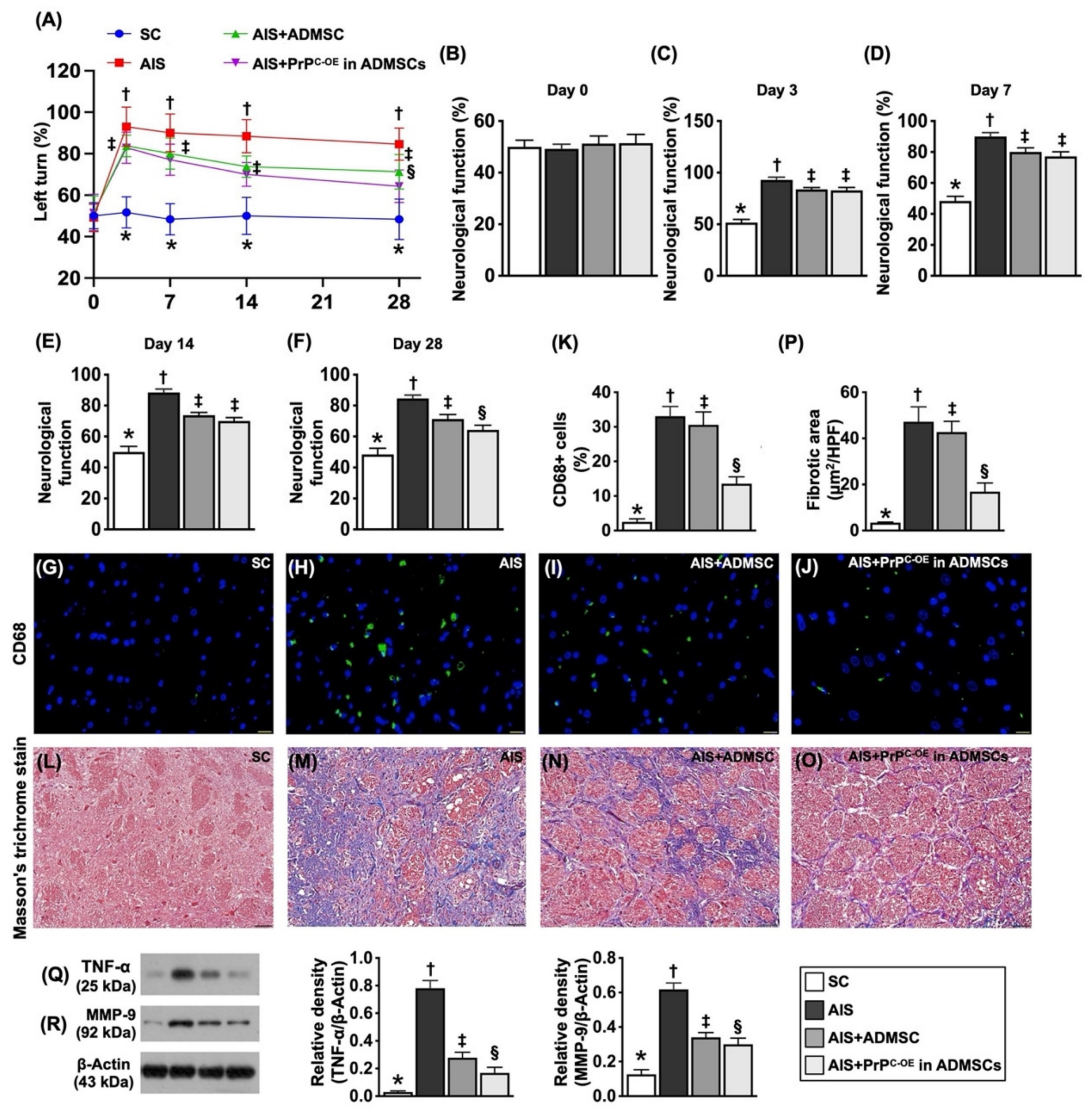
** Time courses of neurological function, inflammation and fibrosis by day 28 after acute ischemic stroke.** Schematically Illustrated the time courses of the neurological function (i.e., corner test). **B)** Neurological function at baseline, p >0.5. **C)** Neurological function at day 3, * vs. other groups with different symbols (†, ‡), *P<*0.0001. **D)** Neurological function ay day 7, * vs. other groups with different symbols (†, ‡), *P<*0.0001. **E)** Neurological function ay day 14, * vs. other groups with different symbols (†, ‡), *P<*0.0001. **F)** Neurological function at day 28, * vs. other groups with different symbols (†, ‡, §), *P<*0.0001. **G to J)** Illustrating the immunofluorescent (IF) microscopic finding (400x) for identification of CD86+ cells (green color). Scale bar in right lower corner represents 20µm. **K)** Analytical result of number of CD68+ cells, * vs. other groups with different symbols (†, ‡, §), *P<*0.0001. **L to O)** Illustrating the immunohistochemical microscopic finding (200x) for identification of fibrotic area (blue color). Scale bar in right lower corner represents 50µm. **P)** Analytical result of fibrotic area, * vs. other groups with different symbols (†, ‡, §), *P<*0.0001. **Q)** Protein expression of tumor necrosis factor (TNF)-α, * vs. other groups with different symbols (†, ‡, §), *P<*0.0001. **R)** Protein expression of matrix metalloproteinases (MMP)-9, * vs. other groups with different symbols (†, ‡, §), *P<*0.0001. All statistical analyses were performed by one-way ANOVA, followed by Bonferroni multiple comparison post hoc test (n=6-10 for each group). Symbols (*, †, ‡, §) indicate significance (at 0.05 level). SC = sham-operated control; IS = ischemic stroke; IS + ADMSCs; IS + PrP^C-OE^ in ADMSCs.
